# Conformational Behavior and Aggregation of Ataxin-3 in SDS

**DOI:** 10.1371/journal.pone.0069416

**Published:** 2013-07-22

**Authors:** Helen M. Saunders, Victoria A. Hughes, Roberto Cappai, Stephen P. Bottomley

**Affiliations:** 1 Department of Biochemistry and Molecular Biology, Monash University, Clayton, Victoria, Australia; 2 Department of Pathology and Bio21 Molecular Science and Biotechnology Institute, The University of Melbourne, Parkville, Victoria, Australia; National Institute for Medical Research, Medical Research Council, United Kingdom

## Abstract

Spinocerebellar ataxia type 3 (SCA3) is one of nine polyglutamine (polyQ) diseases all characterized by the presence of intraneuronal inclusions that contain aggregated protein. Aggregation of ataxin-3, the causative protein of SCA3, has been well characterized *in vitro*, with both pathogenic and non-pathogenic length ataxin-3 undergoing fibrillogenesis. However, only ataxin-3 containing an expanded polyQ tract leads to SCA3. Therefore other cellular factors, not present in previous *in vitro* studies, may modulate aggregation during disease. The interactions between fibrillar species and cell membranes have been characterized in a number of amyloid diseases, including Huntington’s Disease, and these interactions affect aggregation and toxicity. We have characterized the effects of the membrane mimetic sodium dodecyl sulfate (SDS) on ataxin-3 structure and aggregation, to show that both micellar and non-micellar SDS have differing effects on the two stages of ataxin-3 aggregation. We also demonstrate that fibrillar ataxin-3 binds phospholipids, in particular phosphorylated phosphotidylinositols. These results highlight the effect of intracellular factors on the ataxin-3 misfolding landscape and their implications in SCA3 and polyQ diseases in general are discussed.

## Introduction

Ataxin-3 misfolding and its subsequent aggregation underlies the autosomal dominant neurodegenerative disease Spinocerebellar ataxia type 3 (SCA3). This disease is characterized by progressive neuronal dysfunction and the presence of neuronal nuclear inclusions which contain aggregated ataxin-3. The polyglutamine (polyQ) protein ataxin-3 functions as a de-ubiquitinating enzyme, and consists of an N-terminal catalytic Josephin domain which has structural homology to papain-like cysteine proteases, and a comparatively unordered C-terminal region containing two ubiquitin interaction motifs and the polyQ tract [1]–[4]. Expansion of this polyQ tract to greater than 45 residues results in protein aggregation and disease, with the age of onset inversely correlated with repeat length [5], [6].

Knowledge of the kinetic and structural changes involved in ataxin-3 misfolding and aggregation will help us to understand the molecular events and disease progression involved in SCA3. The structural changes and kinetics involved with the *in vitro* aggregation mechanism of ataxin-3 have been characterized. These data indicate that ataxin-3 aggregation involves a two-stage aggregation pathway with interactions facilitated initially by the Josephin domain and subsequently by the polyQ tract [7]–[9]. Despite both the Josephin domain as well as the non-pathogenic length ataxin-3 forming the first stage fibrils *in vitro* [9]–[12], contradicting data exists regarding the presence of non-pathogenic length ataxin-3 aggregates in cells [13]–[15]. However, evidence from various polyQ proteins [16], [17] and model systems [18] increasingly suggests that this multi-stage mechanism is not unique to ataxin-3, and that the flanking regions of the polyQ tract impact upon polyQ aggregation [7], [19], [20].

The intrinsic fibrillogenic nature of both pathogenic and non-pathogenic length ataxin-3 implicates other cellular factors in disease pathogenesis [21]. As a significant proportion of the cellular environment, membranes of varying compositions influence the aggregation of amyloid proteins such as amyloid β-peptide, α-synuclein and prion protein [22]–[24]. Of the polyQ proteins, huntingtin binds various cellular membranes with some evidence that it forms ion channels within bilayer membranes [25]–[27]. Aggregates formed from polyQ peptides are internalized by mammalian cells and cross the cell membrane to gain access to the cytoplasmic compartment [28]. Ataxin-3 has been proposed to associate with cellular membranes in several ways. Within the cell ataxin-3 transiently associates with membranes via its binding partner VCP [29], in addition to directly binding mitochondrial membranes [30]. Interestingly, both huntingtin and ataxin-3 perturb the structure of synthetic lipid bilayers when oligomeric in structure [31], [32], however the impact of membranes and specific lipids on ataxin-3 structure and aggregation is unknown.

Acidic phospholipids, which are present in a number of intracellular membranes, accelerate the aggregation of numerous fibrillogenic proteins including huntingtin [33]–[35]. The detergent Sodium Dodecyl Sulfate (SDS) is an anionic detergent that mimics some characteristics of biological membranes due to its negatively charged head group and long tail. SDS is routinely used as a denaturant [36] and has the ability to induce changes in secondary structure and it can be used to probe the conformational change events occurring during aggregation [37], [38]. The impact of SDS on the aggregation kinetics of amyloidogenic proteins such as β_2_-microglobulin, amyloid-β and α-synuclein has been determined [38]–[40]; however the effects of SDS on polyQ proteins have not been investigated to date.

Using biophysical techniques, we demonstrate that in the presence of SDS, ataxin-3 is able to form aggregates via a number of alternate pathways. We investigate the effects of both micellar and sub-micellar concentrations of SDS on ataxin-3 and show that there are differential effects of SDS at different points of the multi-stage ataxin-3 aggregation pathway. Finally, we show that oligomeric and fibrillar ataxin-3 binds acidic phospholipids, in particular phosphotidylinositols, with different specificities.

## Results

### SDS Increases the α-helical Content in Ataxin-3

**Figure 1:**
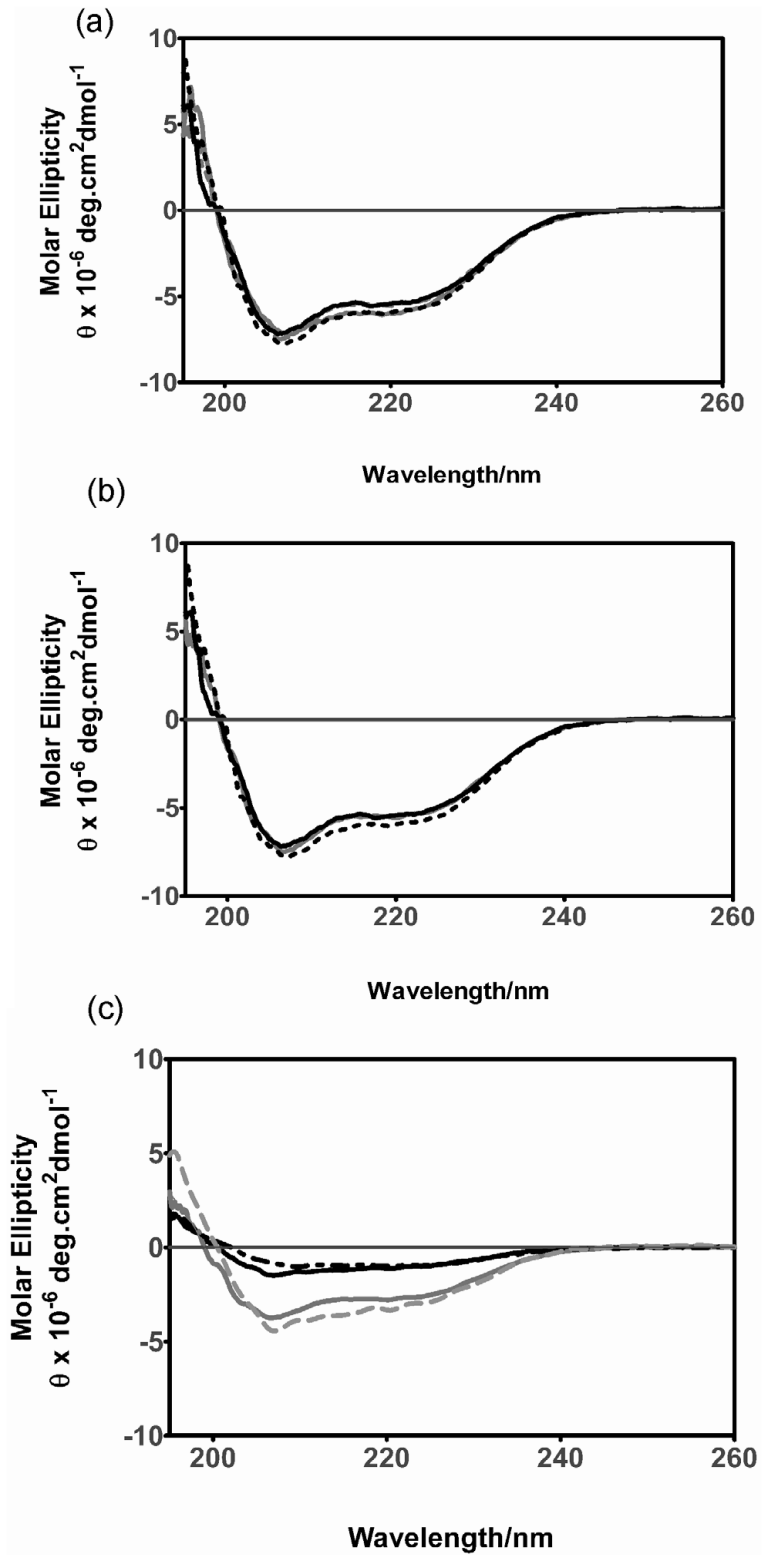
Far-UV CD spectra of ataxin-3 variants in increasing concentrations of SDS. The far-UV CD spectra for (a) ataxin-3(Q64), (b) ataxin-3(Q15) and (c) Josephin were measured at 37°C with increasing concentrations of SDS; 0 mM SDS (*black solid line*), 1 mM SDS (*black dotted line*), 5 mM SDS (*grey solid line)* or 10 mM SDS (*grey dashed line*). The final protein concentration was 30 µM and the spectra measured with a path length of 0.1 mm.

SDS forms micelles at concentrations above the critical micelle concentration (CMC) and in the buffer conditions used within this study the CMC of SDS was determined to be 1.2 mM (data not shown) [38], [41]. SDS has previously been demonstrated to induce helical secondary structure in a range of proteins at concentrations above the CMC [37]–[39]. In this study, the effects of SDS on pathogenic length ataxin-3(Q64), non-pathogenic length ataxin-3(Q15) and the Josephin domain were investigated. Changes in the secondary structure of the ataxin-3 variants with the addition of up to 10 mM SDS were analyzed using far-UV CD spectroscopy (Fig. 1). Consistent with previous reports, all ataxin-3 variants in the absence of SDS displayed spectra with minima at 208 nm and 222 nm, indicative of predominantly α-helical secondary structure [12], [42]–[44]. Only minor changes in secondary structure were observed when SDS was added. In the presence of 1 mM SDS, no significant change in structure occurred for any of the proteins, whereas above 5 mM SDS there was an average increase in α-helical structure of 5% for all proteins (Table 1). The magnitude of the structural changes induced by SDS in ataxin-3(Q64) (Fig. 1A), ataxin-3(Q15) (Fig. 1B) and the isolated Josephin domain (Fig. 1C) were similar, thus suggesting that the changes in secondary structure occur predominantly within the Josephin domain.

**Table 1:** Percentage of α-helical content of monomeric protein with SDS present.

[SDS] mM	Ataxin-3(64)		Ataxin-3(Q15)		Josephin	
	% α- helix	StandardError	% α- helix	Standard Error	% α- helix	Standard Error
0	27.5	2.3	30.3	2.6	30.8	3.5
1	28.7	2.7	30.4	2.0	29.5	1.3
5	32.0	1.9	37.5	2.0	36.4	2.4
10	32.0	1.8	39.1	2.9	35.4	3.3

### SDS Modulates SDS-soluble Aggregation of Ataxin-3

**Figure 2:**
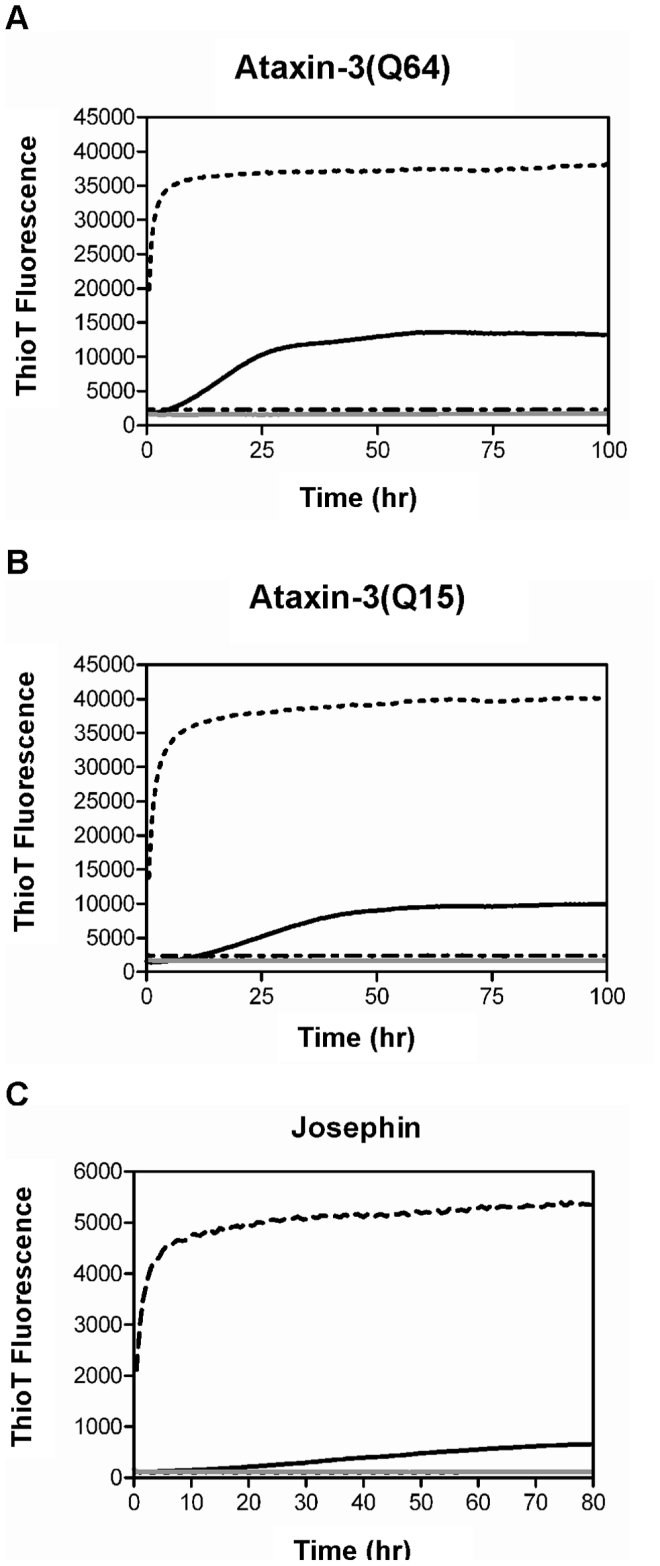
Aggregation of ataxin-3 in the presence of SDS monitored by ThioT. Aggregation of ataxin-3 (30 µM) at pH 7.4 and 37°C in the presence of a range of SDS concentrations was monitored by thioT. ThioT fluorescence values were read at 480 nm (λ_ex_ = 430 nm) every 30 minutes using a fluorescence plate reader. (A) Ataxin-3(Q64), (B) ataxin-3(Q15) and (C) Josephin domain are shown with the addition of 0 mM SDS (*black solid line*), 1 mM SDS (*dashed line*), 5 mM SDS (*grey solid line*) and 10 mM SDS (*dotted and dashed line*).

Ataxin-3 aggregation occurs via a two-stage mechanism. The first stage involves the formation of SDS-soluble curvilinear protofibrils and is common to all ataxin-3 variants. In the second stage of aggregation, only pathogenic length ataxin-3 forms SDS-insoluble fibrils which have a straighter morphology [9]. Formation of the first stage SDS-soluble fibrils was monitored by following changes in thioflavin T (thioT) fluorescence as previously described [9]. Without SDS, all ataxin-3 variants show a sigmoidal aggregation curve indicative of a nucleation-dependent process, with a lag phase followed by exponential growth which then plateaus. The overall aggregation kinetics vary such that the isolated Josephin domain has the slowest aggregation rate and ataxin-3(Q64) the fastest (Fig. 2). The presence of 1 mM SDS eliminated the lag phase of all the ataxin-3 variants, resulting in an immediate exponential growth phase with a rate independent of polyQ length. The midpoint of aggregation decreased to two hours for all proteins with 1 mM SDS present, suggesting that the small conformational change induced by SDS is similar for all proteins and results in a highly amyloidogenic species. In addition, 1 mM SDS also resulted in hyperfluorescence of thioT (Fig. 2) which may be related to a greater number of short fibrils being formed. In contrast, at both 5 mM and 10 mM SDS, there is no increase in thioT fluorescence for any of the ataxin-3 variants, thus suggesting that fibril formation is suppressed at these micellar SDS concentrations. These results, in which a specific range of SDS concentrations around the CMC modulate thioT detected fibrillogenesis, are consistent with those previously reported for a range of other non-polyQ amyloid proteins [37]–[39].

### SDS-insoluble Aggregation of Pathogenic Length Ataxin-3 is Affected by SDS

**Figure 3:**
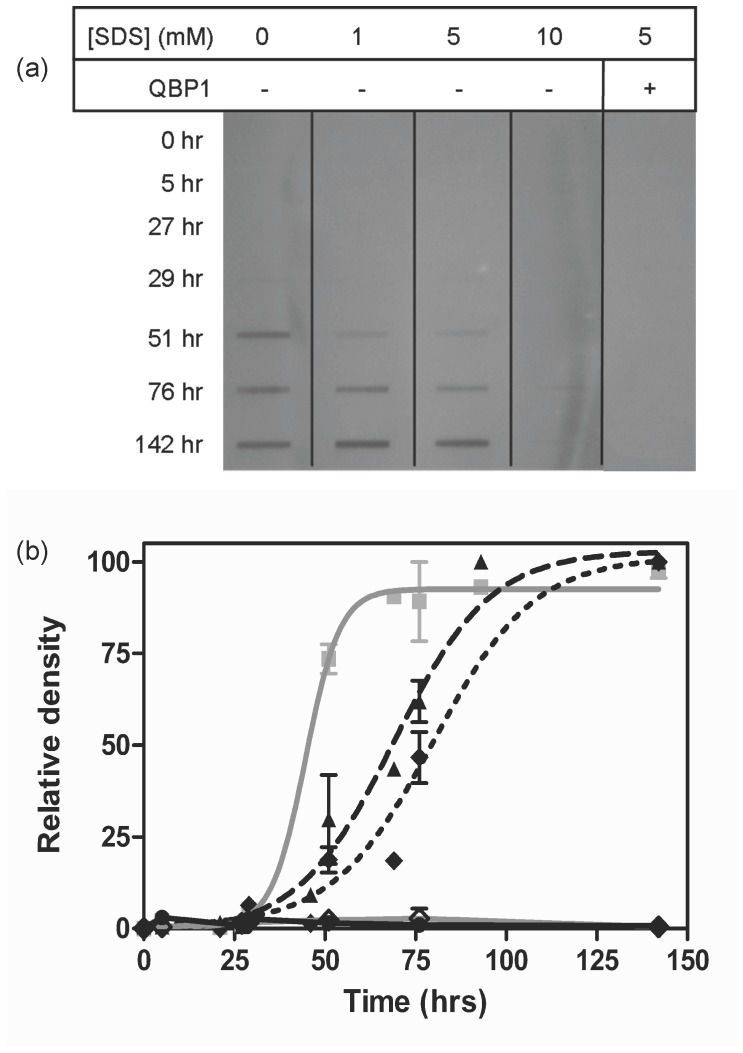
Aggregation of ataxin-3 in the presence of SDS monitored by SDS-insolubility. Formation of SDS-insoluble fibrils was followed by taking aliquots from a 30 µM ataxin-3(Q64) timecourse assay at 37°C, pH 7.4. (A) A representative filter-trap membrane of ataxin-3(Q64) with 0–10 mM SDS is shown. QBP1 was added to a ataxin-3(Q64) containing 5 mM SDS as indicated. (B) Analysis of the filter trap membrane by densitometry. Ataxin-3(Q64) is shown with the addition of 0 mM SDS(-▪-), 1 mM SDS(-▴-), 5 mM SDS (-♦- ), 5 mM SDS with QBP1 (-◊-) and 10 mM SDS (-•-). Results from three independent experiments were fit to an exponential curve.

Having demonstrated that the effects of SDS on the formation of SDS-soluble stage 1 fibrils are similar to those previously reported for other amyloid proteins, we then investigated the effects of SDS on the second stage of the multi-stage ataxin-3 aggregation pathway. The formation of stage 2 aggregates can be monitored using a membrane filter trap assay in which after boiling in 2% (w/v) SDS, only SDS-insoluble fibrils are retained upon the filter. It should be noted that the concentration of SDS used to solubilize the fibrils (2% (w/v) SDS) is six times greater than the concentrations around the CMC which impact upon fibrillogenesis (0.03%–0.29%). It can clearly be seen that in the absence of SDS ataxin-3(Q64) forms SDS-insoluble aggregates following 51 hrs of incubation (Fig. 3A). With 0–10 mM SDS, both non-pathogenic length ataxin-3(Q15) and the Josephin domain did not form SDS-insoluble aggregates when incubated up to 200 hours (data not shown).

Although it was anticipated that due to the sequential nature of the aggregation pathway modulation of stage 1 aggregation would mediate similar kinetic effects on stage 2 aggregation [45], quite different results were obtained. Despite 1 mM SDS significantly accelerating the rate of SDS-soluble fibril formation as detected by thioT fluorescence (Fig. 2A), the formation of SDS-insoluble fibrils by ataxin-3(Q64) with 1 mM SDS is slower and has a midpoint 24 hours later than in the absence of SDS (Fig. 3A,B; Table 2). In contrast, ataxin-3(Q64) with 5 mM SDS shows no increase in thioT fluorescence over 100 hours (Fig. 2A) yet the formation of SDS-insoluble aggregates appear in the filter trap assay from 51 hours (Fig. 3A,B), suggesting that the aggregates are forming on an alternative aggregation pathway. Addition of the polyQ-binding peptide QBP1 eliminated the formation of SDS-insoluble aggregates formed by ataxin-3(Q64) with 5 mM SDS.

**Table 2:** Midpoints of ataxin-3(Q64) aggregation.

	SDS-Soluble Aggregation		SDS-Insoluble Aggregation	
[SDS] mM	Midpoint(hrs)	Standard Error (hrs)	Midpoint (hrs)	Standard Error (hrs)
0	11.50	1.65	44.7	2.7
1	2	0.5	69.0	2.6
5	–	–	79.9	3.0
10	–	–	–	–

In a strongly micellar environment (10 mM SDS), ataxin-3(Q64) does not show the formation of either SDS-soluble (Fig. 2A) or SDS-insoluble aggregates (Fig. 3A,B). Several studies have reported distinct effects of micellar and non-micellar SDS concentrations, with micellar concentrations inhibiting aggregation, and non-micellar concentrations accelerating aggregation [37]–[39]. However with the complexity of the two-stage ataxin-3 aggregation mechanism, there is not such a clear distinction. Only the highly micellar 10 mM SDS inhibits both stages of the pathway, and the other SDS concentrations differentially affect the first and second stages of aggregation.

### SDS Modulates the Change to β-sheet Secondary Structure Typical of Aggregation

With the intriguing formation of thioT unreactive fibrils by 5 mM SDS, we then went on to characterize the changes in secondary structure occurring during aggregation. SDS induces an increase in α-helical structure at concentrations above the CMC (Fig. 1), however a key event in fibrillogenesis is the gain of β-sheet structure, and hence far-UV CD was used to follow the impact of SDS upon this structural transition.

**Figure 4:**
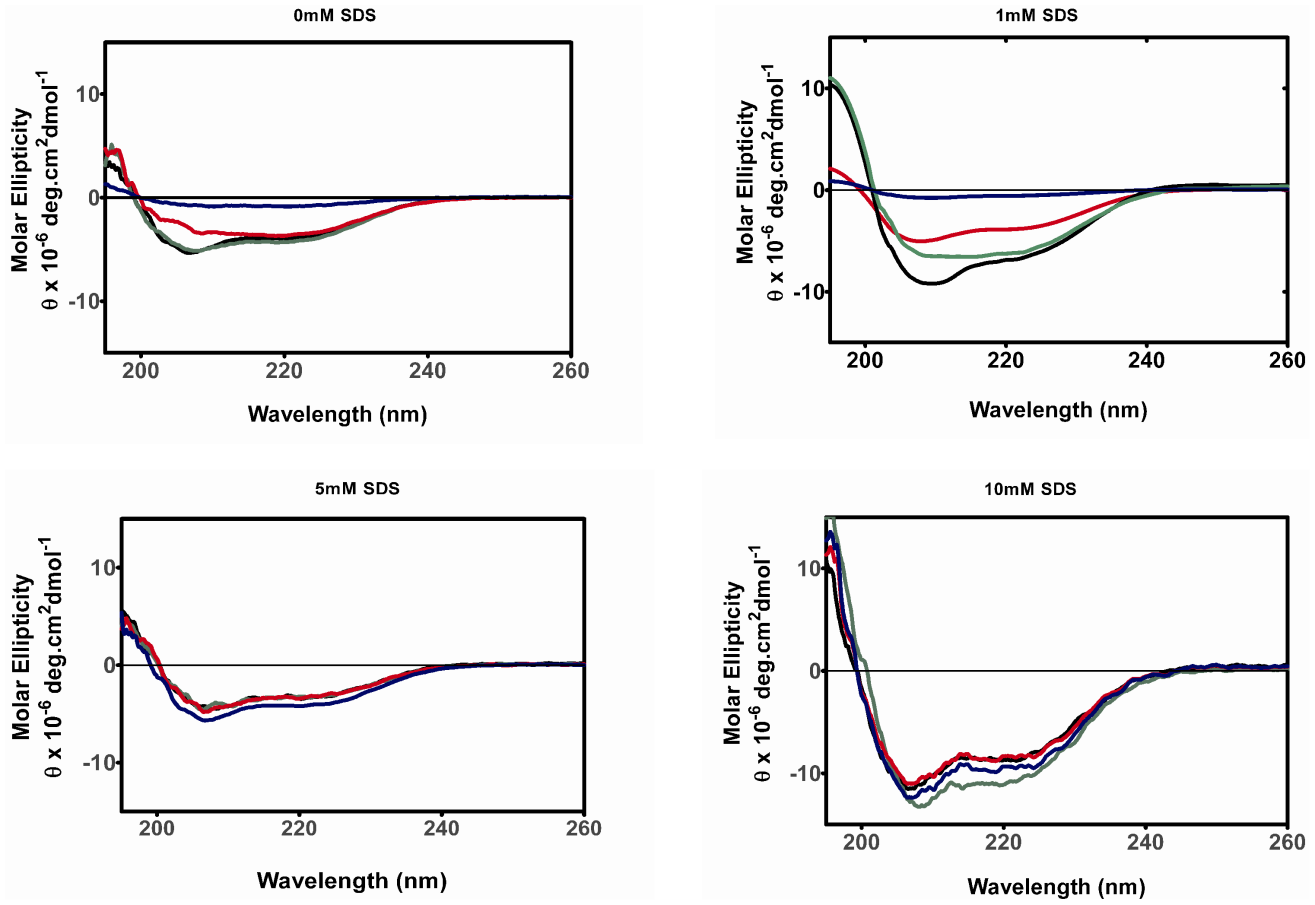
Far–UV CD spectra of ataxin-3(Q64) during aggregation in the presence of SDS. Aliquots of ataxin-3(Q64) were taken from a fibrillogenesis time course assay (see Materials and Methods) and their far-UV CD spectra determined. For each indicated SDS concentration aliquots were taken at times of 0 (*black*), 4 (*green*), 46 (*red*) and 100 (*blue*) hours.

As previously reported, we observed that in the absence of SDS ataxin-3(Q64) converts to a β-sheet rich fibrillar species (Fig. 4A). The loss in signal observed over time has been previously suggested to reflect an increase in light scatter [9]. Incubation in 1 mM SDS (Fig. 4B) accelerates the kinetics of aggregation such that by four hours there has been substantial loss of helical structure and a conversion to β-sheet structure which continues over time with a loss of signal similar to that seen in the absence of SDS at 100 hours (Fig. 4A).

Incubation of ataxin-3(Q64) in both 5 mM and 10 mM SDS leads to a retention of α-helical structure over 100 hours, and for 10 mM SDS there is a small increase in the minima at 208 nm and 222 nm (Fig. 4D). This is consistent with the lack of aggregation detected with 10 mM SDS throughout this study and suggests that SDS has stabilized the α-helical structure to the extent that the conversion to β-sheet is prevented. With 5 mM SDS present, the retention of α-helical structure over time concurs with the lack of thioT fluorescence observed (Fig. 2) and thus suggests that the SDS-insoluble fibrils being formed (Fig. 3A) are more similar to amorphous aggregates than the β-sheet rich amyloid-like fibrils typically formed by ataxin-3. Interestingly, these aggregates are still formed via interactions of the polyQ tract, as addition of QBP1 inhibits their formation (Fig. 3A). The same effects of SDS on the change in secondary structure over time were also observed for ataxin-3(Q15) and the Josephin domain (Fig. S1).

### SDS Addition Results in Changed Morphology of Fibrils

**Figure 5:**
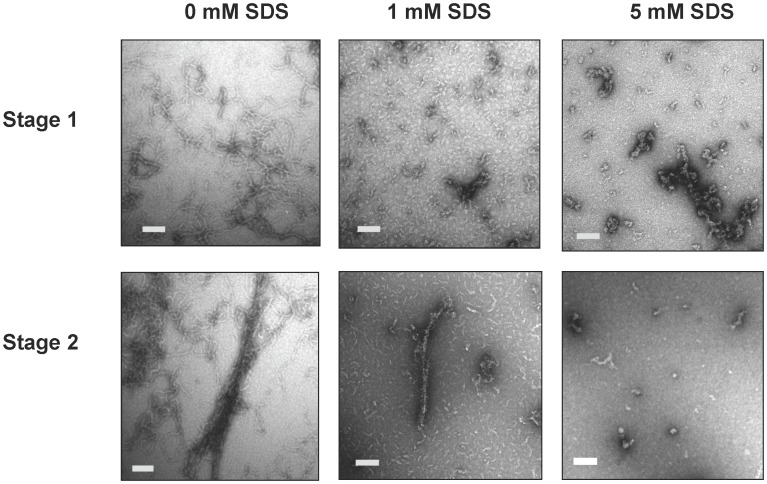
Morphology of fibrils formed by ataxin-3(Q64) with SDS. Transmission Electron Microscopy of ataxin-3(Q64) with 0 mM SDS at 30 hr (A) and 50 hr (D), 1 mM SDS at 5 hr (B) and 30 hr (E) and 5 mM SDS at 30 hr (C) and 50 hr (F). Time course samples from 5 hr, 30 hr or 50 hr were negatively stained using 1% (w/v) uranyl acetate. Scale bars represent 200 nm.

The formation of SDS insoluble fibrils which are not thioT reactive suggests that ataxin-3 is forming these fibrils via an atypical pathway. In order to investigate this further, TEM was used to visualize the morphology of the aggregates being formed with 0, 1, 5 and 10 mM SDS present (Fig. 5, Fig. S2). As expected, ataxin-3(Q64) in the presence of 10 mM SDS did not form fibrils (data not shown). In contrast, with 5 mM SDS ataxin-3(Q64) forms amorphous aggregates with a diameter of 20–30 nm which although SDS-insoluble, do not have a fibrillar morphology but appear clumped together to form large amorphous aggregates of up to 0.5 µm in length (Fig. 5C and F).

The fibrils formed by ataxin-3(Q64) in the presence of 1 mM SDS (Fig. 5B) had lengths of 23–29 nm and a diameter of 5 nm. These fibrils were initially shorter and smaller in diameter than the curvilinear fibrils formed in the absence of SDS which showed diameters of 12–15 nm and lengths of hundreds of nanometers (Fig. 5A) as observed previously[9]. Further incubation resulted in the formation of larger, more rigid SDS-insoluble fibrils which were 40–50 nm in width and up to 1 µm in length (Fig. 5D and E).

### PolyQ Oligomers Interact with Acidic Phospholipids

**Figure 6:**
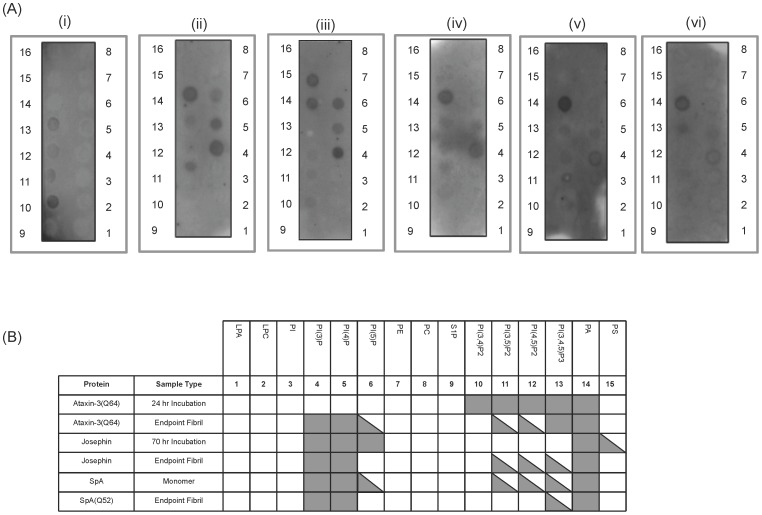
Binding of polyglutamine proteins to phospholipids. (A) Protein-lipid overlays of ataxin-3(Q64) at 24 hrs (i) and 200 hrs (ii), Josephin domain at 70 hrs (iii) and 200 hrs (iv), and monomeric SpA (v) and SpA(Q52) (vi). A representative membrane is shown. (B) A summary of three independent experiments, with a fully shaded square representing strong binding in all experiments, and a triangle representing weak binding in one or two membranes only. Spot 16 is not included as it is a blank dot.

As SDS is a mimetic of acidic phospholipids we then decided to investigate whether ataxin-3 shows a specificity of binding to acidic phospholipids. We incubated ataxin-3(Q64) and the Josephin domain, at specific stages of their aggregation pathway, with a variety of lipids and assessed binding in a protein-lipid overlay assay. Monomeric ataxin-3(Q64) and the Josephin domain both showed no binding to any of the lipids (data not shown). When early time point samples of both proteins were incubated with the PIP strips, binding to phosphorylated phosphotidylinositols (PtdIns) was observed for both ataxin-3(Q64) and the Josephin domain. Interestingly, there were additional lipids which bound to the endpoint fibrils of both the Josephin domain and ataxin-3(Q64) (Fig. 6A i–iv), with the endpoint fibrils binding to essentially the same subset of lipids. Ataxin-3(Q64) incubated with QBP1, an inhibitor of polyQ mediated aggregation, showed the same binding pattern as ataxin-3(Q64) thus suggesting that the lipids are predominantly binding to the misfolded Josephin domain (data not shown).

In order to help confirm that the polyQ tract was not involved, we used the model system Staphylococcus protein A (SpA) with an attached polyQ tract of 52 glutamines (SpA(Q52)) [46]. SpA is a membrane-anchored protein and thus native SpA was used as a control. SpA and SpA(Q52) demonstrated similar binding patterns, further suggesting that the polyQ tract is not involved in lipid binding (Fig. 6 v and vi).

## Discussion

### Effects of SDS on Ataxin-3 Conformation and Aggregation

SDS appears to interact in a common fashion with monomeric fibril-forming proteins, inducing α-helical structure irrespective of the protein’s initial structure [37]–[39], [47]. It is interesting that this can lead to rapid aggregation, considering the key structural change required for aggregation is the conversion to β-sheet dominated structure. A large number of aggregating proteins, including both disease-causing proteins and the fibril-forming peptide SRC3 show this accelerated aggregation at sub-micellar SDS concentrations, and slowed or inhibited aggregation at SDS concentrations well above the CMC [37]–[40], [48]. The inhibition of aggregation may be due to the highly α-helical structure stabilized by micellar SDS concentrations (Fig. 1) preventing the transition to β-sheet structure which occurs during aggregation. Alternatively the inhibition of aggregation may be a result of steric and/or electrosctatic effects contributed by the micelles that prevent proteins which are interacting with the micelle from interacting and aggregating. For ataxin-3 at sub-micellar concentrations, there was significant acceleration of SDS-soluble aggregation, similar to other fibrillogenic proteins, in addition to a significant increase in thioT fluorescence intensity. The molecular basis for this dramatic acceleration is unknown however one possibility is that the SDS is destabilizing the native form of the protein which allows it to access more non-native conformations some of which may be prone to aggregation. A similar behavior has been reported before with low concentrations of denaturant [10], [44].

**Figure 7:**
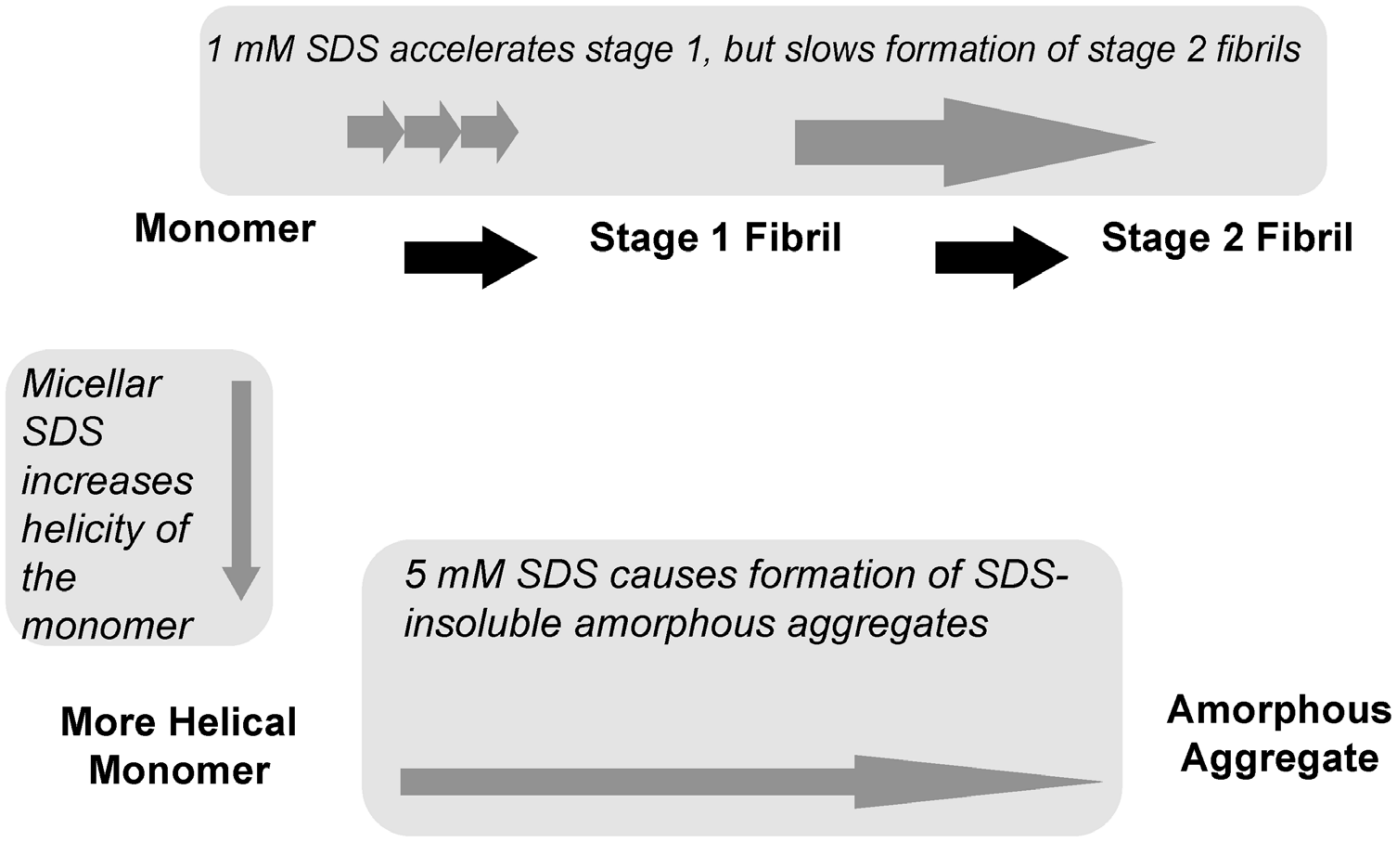
Summary of effects of SDS on ataxin-3 aggregation. Schematic summarizing the effects of micellar and non-micellar SDS on both stages of ataxin-3 aggregation.

An interesting result in this study was the differing effects of SDS upon formation of SDS-insoluble ataxin-3 fibrils as opposed to the more commonly observed effects of SDS on SDS-soluble fibril formation. With 1 mM SDS, the lack of an observable lag phase suggests either the lag phase has been accelerated to the extent that it cannot be observed or that ataxin-3 is following an alternate mechanism not involving nucleated elongation. Although the subsequent formation of SDS-insoluble aggregates is slower, the morphology of the end point aggregates remains unchanged. Thus it appears that with sub-micellar SDS present, the protein is proceeding through the typical aggregation pathway, with differing rates of stage 1 and stage 2 aggregation (Fig. 7).

In contrast to 1 mM SDS, the morphology of the aggregates is not fibrillar in the presence of 5 mM SDS, thus demonstrating that ataxin-3 has the ability to undertake alternate aggregation pathways to form a range of fibril types (Fig. 7). This is supported by the lack of both thioT binding and conversion to β-sheet structure. At this micellar concentration, although there is no formation of thioT reactive SDS-soluble aggregates, SDS-insoluble aggregates are still formed. These aggregates have a substantially different morphology, appearing amorphous in structure, however they are still formed via interactions of the polyQ tract, as formation of these aggregates is inhibited by QBP1 (Fig. 3A). The formation of different aggregate morphologies is not unprecedented as environmental conditions affect the type of aggregate formed by a number of proteins *in vitro* [49], [50]. Within the cell such changes in the intracellular environment could be achieved by conditions of stress, such as elevated temperature or decreased pH, or changes in membrane composition [34], [51].

Ataxin-3 oligomers and fibrils displayed a specificity in binding to PtdIns with varying degrees of phosphorylation. PtdIns are generally located on the cytoplasmic side of the plasma membrane and are present in specific membranes depending on phosphorylation, with a higher abundance of these lipids (10%) in brain tissue [52]. Although monomeric huntingtin also bound similar phospholipids [33], it appears that this is not a common polyQ specific effect as only fibrillar species of ataxin-3 showed binding. Furthermore, when the polyQ-binding peptide QBP1 was added there was no change to the binding pattern which suggests that binding occurs through the Josephin domain. This is similarly seen in the SDS experiments in this study, where the effect of SDS on the Josephin domain is identical to that on ataxin-3, and unaffected by QBP1.

Phospholipids have been demonstrated to affect aggregating proteins by creating regions which have a local environment with a decreased pH, and through electrostatic interactions which can increase the local concentration of protein at the membrane and induce partial unfolding of proteins [53]–[55]. It is interesting that oligomers and fibrillar ataxin-3 bound to the lipid overlay with different specificities as several studies show that oligomers have a generic ability to permeabilize cell membranes by creating pores or single channels within membranes [56]–[58].

Overall, our findings demonstrate the sensitivity of ataxin-3 fibril formation to solution conditions and suggest a possible role for lipid molecules in the development of SCA3. The specificity of binding with only fibrillar species associating with phosphorylated phospholipids provides a link between ataxin-3 and the growing evidence that soluble oligomers disrupt membranes as part of the mechanism of toxicity within amyloidoses [47], [59], [60].

## Materials and Methods

### Materials

Phenylmethylsulfonyl fluoride, β-mercaptoethanol and thioT were all obtained from Sigma.

### Expression and Purification of Ataxin-3 variants

All ataxin-3 variants were expressed and purified as previously described [61], [62], and the proteins were stored at −80°C. Following purification the deubiquitinating activity of the proteins was measured [10] and before use all were analyzed using gel filtration to ensure that no multimeric species were present.

### Circular Dichroism

Far-UV CD spectra were measured on a Jasco-810 spectropolarimeter at 37°C using a path length of 0.1 mm. Scans of monomeric protein in the presence of different concentrations of SDS were carried out. For time course assays, protein aliquots were diluted 1∶1 with TBSG (100 mM Tris, 80 mM NaCl, 10% (v/v) glycerol, pH 7.4), to a final concentration of 30 µM protein. In all scans, spectra were measured from 190 to 260 nm with a scanning speed of 50 nm/min. The CD spectra were analyzed by spectral deconvolution using the CONTINLL algorithm [63].

### Calculation of the CMC of SDS

To calculate the CMC, the fluorescent probe ANS was used as previously described [38]. The fluorescence intensity was measured at 475 nm, with an excitation wavelength of 385 nm using a PerkinElmer Life Sciences LS50B spectrofluorometer with a thermostatted cuvette holder and a 1 cm pathlength quartz cuvette. The reaction mix contained 10 µM ANS with 0–10 mM SDS in TBSG, pH 7.4 at 37°C. Two straight lines were fit to the data points with the intersection determining the CMC.

### Fibrillogenesis Time Course Assays

Ataxin-3 variants (30 µM) were incubated in TBSG containing 5 mM EDTA, 15 mM β-mercaptoethanol and 2 mM phenylmethylsulfonyl fluoride, with the addition of 0–10 mM SDS as required. Samples were incubated without shaking at 37°C in air-tight containers to prevent evaporation.

### Thioflavin T Fluorescence

ThioT fluorescence measurements were recorded on a Gemini platereader, using the buffering conditions described previously with the addition of 30 µM ThT and 0–10 mM SDS. Excitation and emission wavelengths of 430 nm and 480 nm with a cut off filter of 455 nm were used, and both excitation and emission were read from the underneath of a black clear bottom plate. All reactions were completed at 37°C with no shaking.

### Membrane Filter Trap Assay

Aliquots containing 7.4 µg of protein were taken from the fibrillogenesis reaction, diluted 1∶1 with a 4% (w/v) SDS/100 mM DTT solution and then boiled for 5 min at 100°C. 200 µL of 2% (w/v) SDS was then added to each of the samples, and 5 µg of protein were filtered through a 0.2 µm cellulose acetate membrane (Schleicher and Schuell) using a Bio-Rad Bio-Dot SF microfiltration unit. The membrane was then washed twice by filtering 200 µL of 0.1% (w/v) SDS, and blotted with a hexahistidine (His6) antibody (Serotec). Densitometry was completed using the program Phoretix 1D Quantifier.

### Transmission Electron Microscopy

TEM images were obtained using a Hitatchi H7500 transmission electron microscope with an accelerating voltage of 80 kV. The samples were adsorbed onto a carbon-coated grid and stained with 1% (w/v) uranyl acetate.

### Protein-lipid Overlay

The protein-lipid overlay assay was performed using fibrillar protein stopped at various timepoints. The protein was incubated with the phospholipids (PIP Strips, Molecular Probes) overnight at 4°C. Each lipid dot contained 100 pmol of phospholipids. The membrane was then blotted using His6 primary antibody (Serotec) and peroxidase-conjugated secondary antibody as described [64].

## Supporting Information

Figure S1 **Far-UV CD spectra of ataxin-3(Q15) and the Josephin domain during aggregation in the presence of SDS.** Protein aliquots were taken from a fibrillogenesis time course assay and the far-UV CD spectra determined. For each indicated SDS concentration aliquots were taken at times of 0 hr (black), 4 hr (green), 46 hr (red) and 100 hr (blue).
(TIFF)

Figure S2 **Morphology of fibrils formed by ataxin-3(Q15) and Josephin with SDS.** Transmission Electron Microscopy of ataxin-3(Q15) with 0 mM (A), 1 mM (B) and 5 mM SDS (C), and Josephin domain with 0 mM (D), 1 mM (E) and 5 mM (F) SDS. Samples after 100 hr incubation were negatively stained using 1% (w/v) uranyl acetate. Scale bars represent 200 nm.
(TIFF)
