# Supplementary figures and images for: Conformational Behavior and Aggregation of Ataxin-3 in SDS
Source: PLoS One. 2013 Jul 22;8(7):e69416. doi: 10.1371/journal.pone.0069416 (PMC3718759; doi:10.1371/journal.pone.0069416)

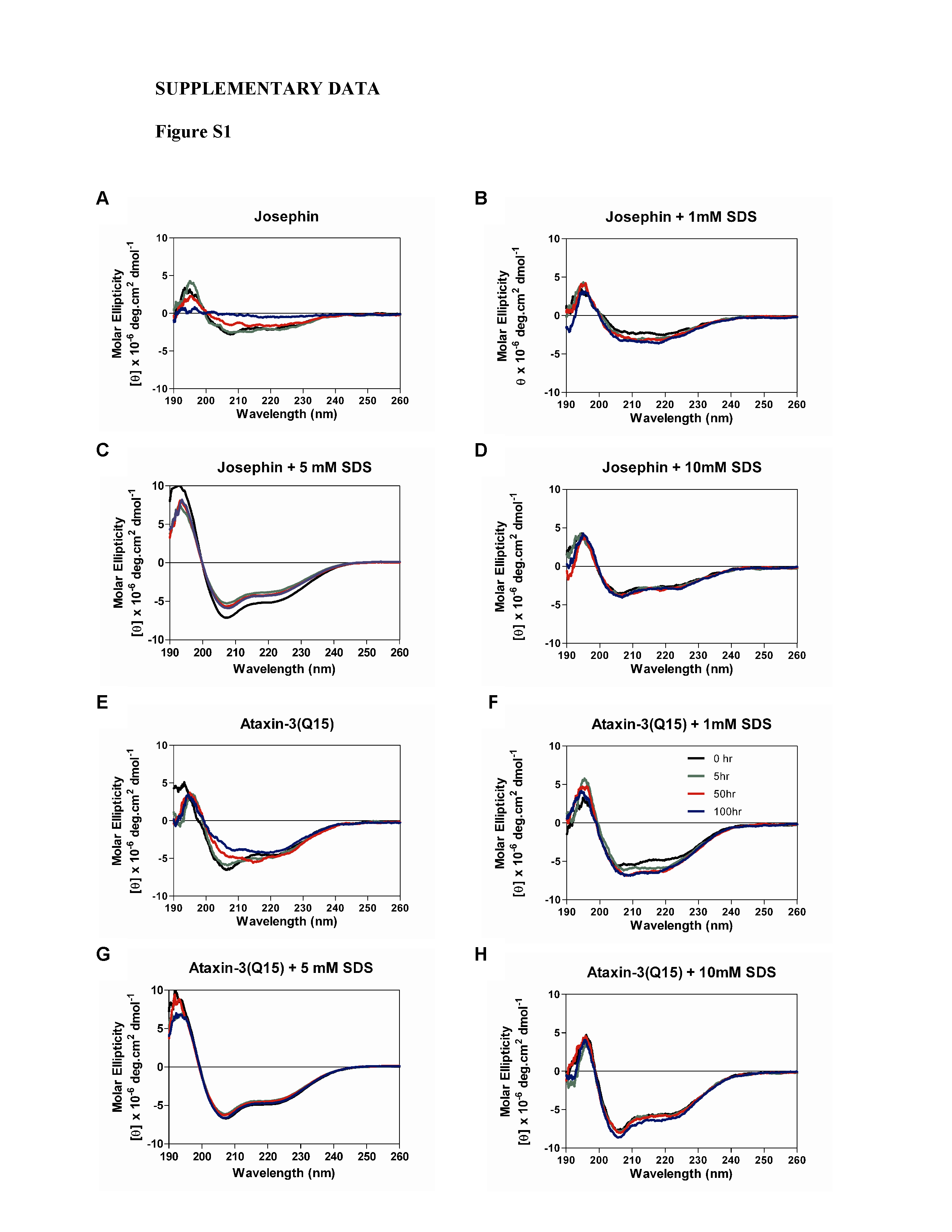

Supplement: Figure S1 — Far-UV CD spectra of ataxin-3(Q15) and the Josephin domain during aggregation in the presence of SDS. Protein aliquots were taken from a fibrillogenesis time course assay and the far-UV CD spectra determined. For each indicated SDS concentration aliquots were taken at times of 0 hr (black), 4 hr (green), 46 hr (red) and 100 hr (blue). (TIFF) [file pone.0069416.s001.tiff]

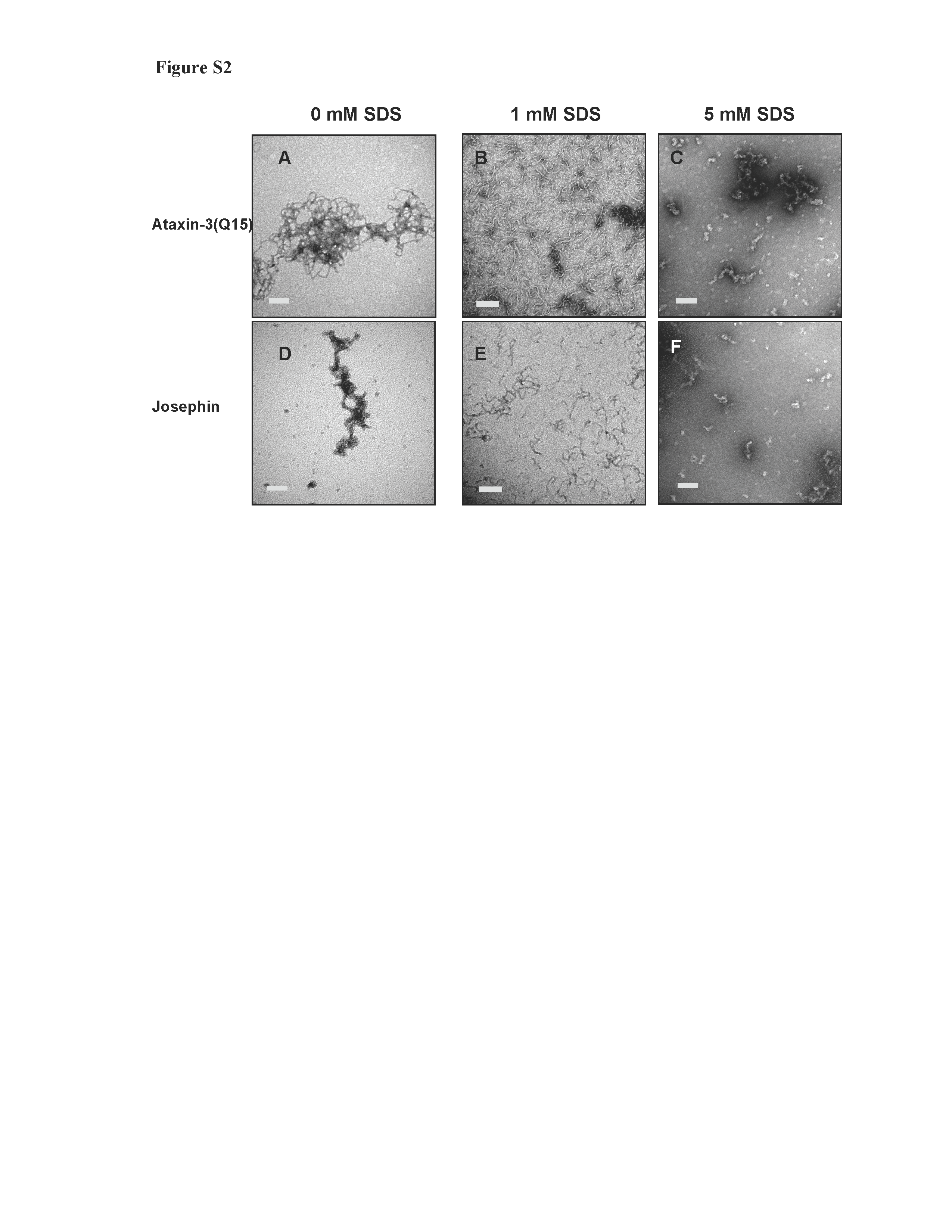

Supplement: Figure S2 — Morphology of fibrils formed by ataxin-3(Q15) and Josephin with SDS. Transmission Electron Microscopy of ataxin-3(Q15) with 0 mM (A), 1 mM (B) and 5 mM SDS (C), and Josephin domain with 0 mM (D), 1 mM (E) and 5 mM (F) SDS. Samples after 100 hr incubation were negatively stained using 1% (w/v) uranyl acetate. Scale bars represent 200 nm. (TIFF) [file pone.0069416.s002.tiff]
